# Elevated Antigen-Driven IL-9 Responses Are Prominent in Peanut Allergic Humans

**DOI:** 10.1371/journal.pone.0045377

**Published:** 2012-10-11

**Authors:** Jungang Xie, Larisa C. Lotoski, Rishma Chooniedass, Ruey-Chyi Su, F. Estelle R. Simons, Joel Liem, Allan B. Becker, Jude Uzonna, Kent T. HayGlass

**Affiliations:** 1 Department of Immunology, University of Manitoba, Winnipeg, Canada; 2 Department of Pediatrics and Child Health, University of Manitoba, Winnipeg, Canada; 3 Windsor Allergy Asthma Education Centre, Ontario, Canada; McGill University, Canada

## Abstract

Food allergies, and peanut allergy in particular, are leading causes of anaphylactic fatalities worldwide. The immune mechanisms that underlie food allergy remain ill-defined and controversial, in part because studies in humans typically focus on analysis of a limited number of prototypical Th1/Th2 cytokines. Here we determine the kinetics and prevalence of a broad panel of peanut-driven cytokine and chemokine responses in humans with current peanut allergy vs those with stable, naturally occurring clinical tolerance to peanut. Our primary focus is identification of novel indicators of immune dysregulation. Antigen-specific cytokine mRNA and protein responses were elicited in primary culture via peanut or irrelevant antigen (*Leishmania* extract, milk antigens) mediated stimulation of fresh peripheral blood cells from 40 individuals. Peanut extract exposure in vitro induced a broad panel of responses associated with Th2/Th9-like, Th1-like and Th17-like immunity. Peanut-dependent Type 2 cytokine responses were frequently found in both peanut allergic individuals and those who exhibit clinical tolerance to peanut ingestion. Among Th2/Th9-associated cytokines, IL-9 responses discriminated between allergic and clinically tolerant populations better than did commonly used IL-4, IL-5 or IL-13 responses. Comparison with responses evoked by unrelated control antigen-mediated stimulation showed that these differences are antigen-dependent and allergen-specific. Conversely, the intensity of IL-12, IL-17, IL-23 and IFN-γ production was indistinguishable in peanut allergic and peanut tolerant populations. In summary, the ability to generate and maintain cytokine responses to peanut is not inherently distinct between allergic and peanut tolerant humans. Quantitative differences in the intensity of cytokine production better reflects clinical phenotype, with optimally useful indicators being IL-9, IL-5, IL-13 and IL-4. Equivalent, and minimal, Ag-dependent pro-inflammatory cytokine levels in both healthy and peanut allergic volunteers argues against a key role for such cytokines in maintenance of clinical tolerance to food antigens in humans.

## Introduction

Food allergies, and peanut allergy in particular, are leading causes of anaphylaxis. While their natural history, epidemiology and social consequences are increasingly well defined [1]–[4], validated gains in diagnosis, prevention or management are generally viewed as modest [5]–[7]. One key roadblock hindering development of improved prophylactic and therapeutic interventions is our limited understanding of which immune responses are differentially expressed in humans with food allergy vs those exhibited by individuals who maintain clinical tolerance to common foods.

Several factors contribute to this challenge. Logistical difficulties associated with eliciting, detecting and quantifying food antigen-driven cytokine responses in humans using primary culture often leads investigators to make use of polyclonal activators as surrogates for biologically relevant ligands, notwithstanding the fact that such approaches lead to activation of a much larger proportion of the immune repertoire than does antigen-dependent activation. Secondly, despite recognition of the large number of cytokines involved in immune regulation, the research focus in human food allergy research to date has primarily been on a rather restricted panel of cytokines. IFNγ plus IL-5 and/or IL-13 as indicators of Th1-like and Th2-like responses are frequently the main or only readouts examined. While Th2-skewed Ag-specific responses are obviously common to many allergic disorders, identification of or comparison between other Th1 or Th2-immunity cytokines, or those linked to Th9 or Th17-like phenotypes remains speculative in humans. With a broad variety of innovative therapeutic approaches under consideration to modulate hypersensitivity [8]–[12], better understanding of characteristic human cytokine responses to common foods is key to progress. Murine models provide excellent tools, but with few exceptions [13], [14], most require adjuvant and have other characteristics that may limit their ability to directly extrapolate their findings.

Here, we utilized short term antigen-stimulated primary culture of peripheral blood mononuclear cells (PBMC) directly ex vivo to assess peanut dependent cytokine and chemokine production in humans of distinct clinical phenotypes. Experimental conditions were identified to allow examination of a broad panel of cytokine mRNA targets that we hypothesized are differentially expressed in peanut allergic humans versus in those humans who exhibit sustained naturally occurring clinical tolerance to peanut. We map the kinetics of mRNA expression for this panel of established and novel Th2-associated (IL-4, IL-5, IL-9, IL-13), proinflammatory/Th17 related (IL-17, IL-23) and Th1-immunity associated cytokine (IFNγ, IL-12, IL-23) responses in humans following acute in vitro re-stimulation with peanut, then apply this strategy to 40 clinically well characterized individuals. The data reveal that differences in food-Ag driven IL-9 mRNA and protein responses are superior to most extensively used, “classical” Th2 markers. Peanut dependent IL-17 mRNA responses are frequent and intense but do not differ between clinical phenotypes. Indeed, none of the Th1 or Th17-associated indicators examined revealed differential expression of pro-inflammatory cytokine gene expression between the two populations. Collectively, the data reveal ubiquitous CD4 T cell dependent responses to peanut allergen in the population as a whole, identify novel cytokine responses and reveal quantitatively distinct patterns of peanut Ag-driven responses between peanut allergic individuals and those who maintain clinical tolerance to this common food antigen. The findings argue for their utilization in future investigations of the complex mechanisms that underlie food allergy vs clinically apparent, functional tolerance.

## Materials and Methods

### Subjects

Following University of Manitoba Research Ethics Board approval, written informed consent was obtained before enrollment. Twenty peanut allergic and 20 individuals exhibiting clinical tolerance to peanut were recruited. Peanut allergic individuals had (i) convincing histories of anaphylaxis (defined [6] as a serious allergic reaction that may cause death taking place within the previous five years despite stringent efforts to avoid dietary peanut, (ii) clearly positive epicutaneous tests via the prick-through drop technique using 1∶20 w/v peanut/glycerin extract, (Omega Laboratories, Montreal Canada) and (iii) elevated peanut specific IgE ImmunoCAP results. Several of these peanut allergic individuals exhibited positive skin tests, clinical symptoms to additional allergens, allergic rhinitis and/or asthma. Peanut non-allergic individuals were recruited on the basis of (i) a detailed allergy history consistent with a lack of clinical reactivity to peanut, ever, (ii) negative epicutaneous tests (<3 mm wheals) and (iii) peanut specific IgE ImmunoCAP results <0.35 kU/L. These participants took no precautions to avoid peanut, and ingested it without developing any symptoms or signs. Table 1 provides more detailed information on these populations. Due to local research ethics board constraints, screening for individuals exhibiting peanut sensitization alone was not possible.

**Table 1:** Demographic and Clinical Characteristics of Participants.*

	Peanut allergic	Peanut tolerant
No. of individuals	20	20
Males	14/20	11/20
Age (y)	18.6 (2–54)	21.1 (12–33)
Wheal diameter (mm)	9.2 (4–20)	0 (0)
Peanut specific IgE (kU_A_/L)	17.3 ( 0.5–63)	<0.35 (<0.35)
Other food allergy	11/20	1/20
Asthma	12/20	1/20
Allergic rhinitis	15/20	4/20

* Values are expressed as median (range) or absolute numbers within the population.

### Peripheral blood mononuclear cell isolation and culture

All studies were carried out with fresh samples directly ex vivo. 20–50 ml whole blood was collected into 2 ml 2.7% EDTA. Whole peanut antigen extract was prepared from roasted peanut extracted for 72 h in aqueous buffer (borate pH 8.4) with the protein concentration determined by BCA Protein Assay (Pierce, Rockford, IL). Endotoxin levels of complete medium and peanut antigen were assessed by LAL assay (Associates of Cape Cod, East Falmouth MA). All reagents and antigens used exhibited levels <0.01 EU/ml in culture.

Culture systems were established and optimized using peanut extract titrated in culture from 10–200 µg/ml. Briefly, fresh PBMC were cultured in 10% pre-selected FBS-RPMI containing L-glutamine and antibiotic/antimycotic at 5×10^5^/well in 96 well round bottom plates with 6 replicate wells for each time point harvested. In independent experiments, Ag-specificity requirements for cytokine production were assessed using SLA (soluble *Leishmania* antigen, 10 µg/ml) or semi-purified milk antigens. SLA was prepared as described [15] with minor modifications. Briefly, 7-day stationary phase promastigotes were washed in cold PBS, sonicated and viewed microscopically to ensure that all parasites were disrupted. The parasite lysate was then centrifuged, dialyzed against PBS, sterilized by passage through a 0.22 um filter and stored at −80 C. Whole bovine casein ( a mixture of alpha-S1-casein, alpha-S2-casein, beta-casein, k-casein) and whey ( a source of α-lactalbumin and β-lactoglobulin) were isolated from fresh milk as described [16] and used for stimulation at final protein concentrations of 100 to 900 ug/ml. Anti-CD80 and anti-CD86 Abs (BDBiosciences) were added to some cultures at a final concentration of 5 ug/ml each to assess costimulatory requirements for IL-9 production.

Culture supernatants were collected and stored at −20 C until analyzed by ELISA while cell pellets were stored at −80 C in RNAlater (Qiagen) until analysis. Unstimulated time 0 samples were also prepared from 3–5×10^6^ PBMC directly ex vivo.

### RNA isolation, Reverse Transcription and Quantitative PCR

Total RNA was isolated from PBMC. cDNA was prepared with first strand synthesis using random hexamers and SuperScript III reverse transcriptase (Invitrogen, Carlsbad, CA). The reaction mix was incubated at 25°C for 5 minutes, 42°C for 1.5 hours for reverse transcription, and finally at 85°C for 5 minutes for reverse transcriptase inactivation. mRNA levels of the cytokines examined were determined by Q-PCR (Applied Biosystems, USA) using primers we designed and validated (Table S1). PCR products were detected in 96-well plates in duplicate using SYBR Green Master Mix (Stratagene, CA). For each cytokine, the “fold change” in mRNA expression was calculated using Delta-Delta Ct Analyses (ABI Data Analysis Software) with 18 s rRNA as for input normalization and paired unstimulated cells as comparison point for each stimulated sample.

### Cytokine ELISA

Primary culture supernatant cytokine levels were determined with reagents from BioLegend (San Diego, CA) for IL-5, IL-9, IL-13 and IFNγ as described [17], [18]. Sensitivity was 5.86, 9.38, 5.86 pg/ml and 0.625 IU/ml, respectively, with inter-assay variability generally of 5–10%.

### Statistical analysis

Analyses were carried out with Prism 5 software (GraphPad, San Diego, CA). Where data sets exhibited a Gaussian distribution, Student's t tests were used with matched or unmatched pairs as appropriate. Nonparametric analyses were conducted with validated Wilcoxon matched or unmatched pairs, as appropriate. Two-tailed P values are reported, with differences considered significant if p<0.05.

## Results

### Optimization of peanut antigen concentration and kinetics of cytokine production by fresh PBMC

The twenty peanut allergic individuals studied exhibited convincing clinical histories of anaphylaxis within the previous 5 years, strong epicutaneous tests to peanut challenge (mean 18.6 mm wheal diameter) and elevated peanut specific IgE levels (9.2 kU/L). The 20 control individuals with clinical tolerance to peanut had a reported lack of clinical reactivity to peanut ever, negative epicutaneous tests, negative peanut specific IgE ImmunoCAP results and regular dietary peanut exposure (Table 1).

To identify optimal experimental conditions to elicit and quantify Ag-stimulated human cytokine mRNA responses for the range of cytokines being examined, freshly isolated PBMC were cultured without stimulation and in the presence of a range of low endotoxin peanut antigen extract concentrations. PBMC were used in preference to highly enriched CD4 T cell populations because multiple cell types, including but not limited to CD4 T cells, contribute to the net cytokine response evoked by allergen exposure. mRNA levels of representative cytokines associated with Th1, Th2 and Th17-like immune responses were quantified after 3 h, and again at days one through six of culture. No statistically significant differences were observed between the three antigen concentrations in their capacity to elicit responses, so the 100 ug/ml peanut concentrations were used for all subsequent experiments. Peanut allergic individuals exhibited indistinguishable kinetics from those with clinical tolerance to peanut. Illustrative data from three representative experiments of 12 performed are shown in Figure S1.

**Figure 1:**
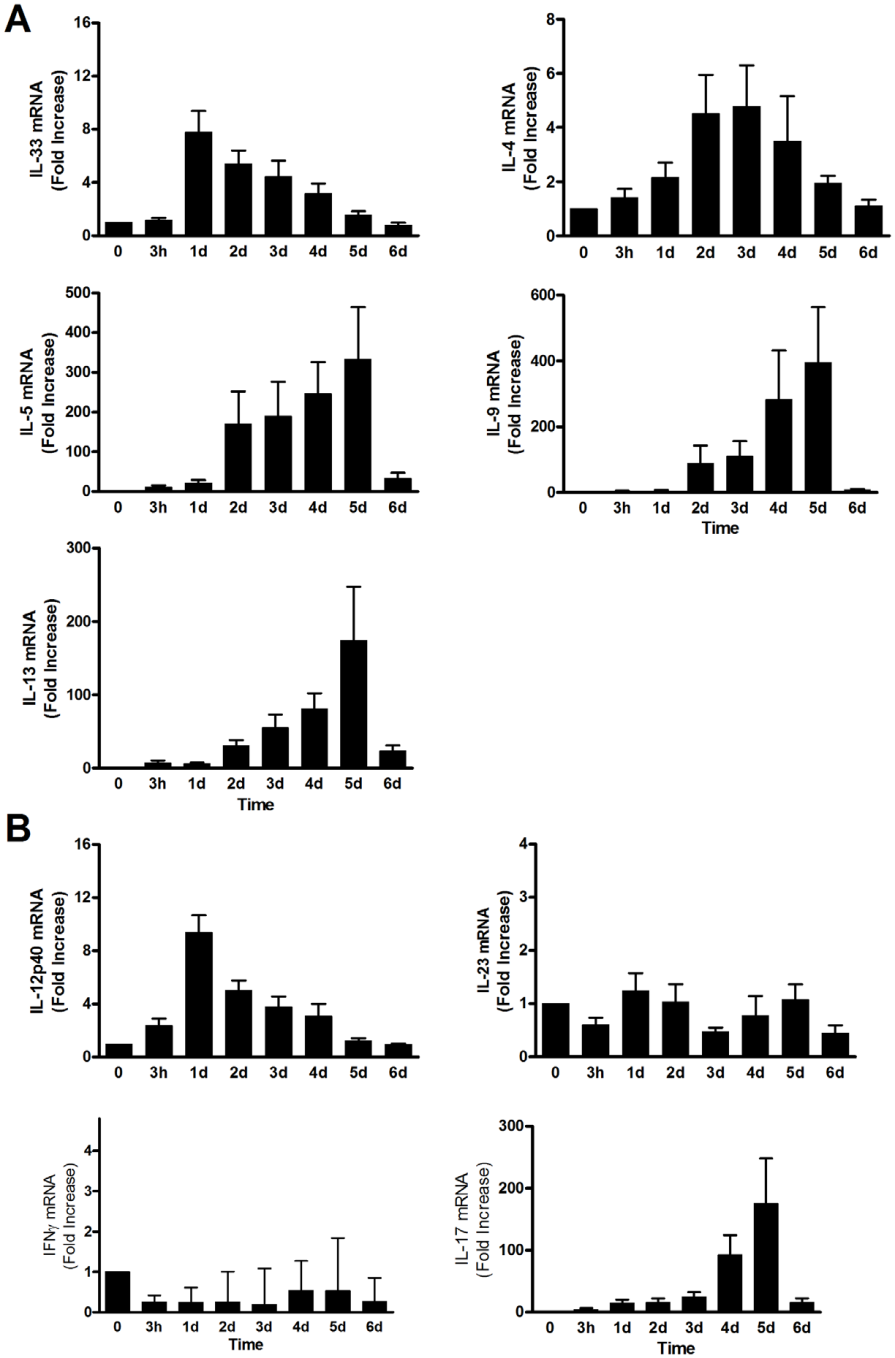
Kinetic characterization of peanut antigen stimulated cytokine mRNA responses. PBMC from 12 individuals were cultured with 100 µg/ml peanut Ag with cells then harvested at each time point shown. Results are expressed as mean (±SEM) fold changes in mRNA levels normalized against un-stimulated controls. **Panel A:** Th2/Th9 associated cytokines. **Panel B:** Th1/Th17-associated cytokines.

Recognizing that Q-PCR analysis only increases the sensitivity of detection if paired with detailed knowledge of expression for the genes of interest, we individually determined kinetics for each cytokine to be examined. Peak IL-33 mRNA responses to peanut antigen were exhibited at d.1 and IL-4 at d.3 whereas IL5, IL-9 and IL-13 were maximal at d.5 (Figure 1). Among pro-inflammatory Th1 and Th17 associated cytokines, IL-12/23 p40 expression peaked at day 1 whereas IL-23 p19, IL-12 p35 and IFNγ levels did not exhibit increases in response to peanut stimulation at any of the times studied. IL-17A mRNA was strongly stimulated upon peanut antigen exposure, peaking at d.5. These optimum time points were used for all subsequent analyses.

Given the extensive diversity in human versus inbred murine populations, hence the inherent challenges in identifying differences between highly heterogeneous populations, it was important to assess the extent of experimental reproducibility in QPCR within individuals. Inter-assay reproducibility of QPCR data are not frequently reported. To assess variance, mRNA from multiple samples were independently reanalyzed in separate experiments. Values agreed closely, demonstrating the reproducibility of the technique employed and the data obtained (Table S2). Similarly, while inter-individual variation in the intensity of responses was substantial between individuals, characteristic of most immune responses in human populations, differences in the kinetics of production in different individuals for each of the cytokines examined was minimal. Collectively, these data indicate that while it is clearly essential to examine each cytokine at the time of its peak expression (rather than a single arbitrary time point for logistical convenience), optimal Ag-driven production of, for instance, IL-4 and IL-13 was consistently at days 3 and 5 respectively, regardless of the participant examined or their clinical status.

### Differential cytokine mRNA expression in peanut allergic vs clinically tolerant individuals

**Figure 2:**
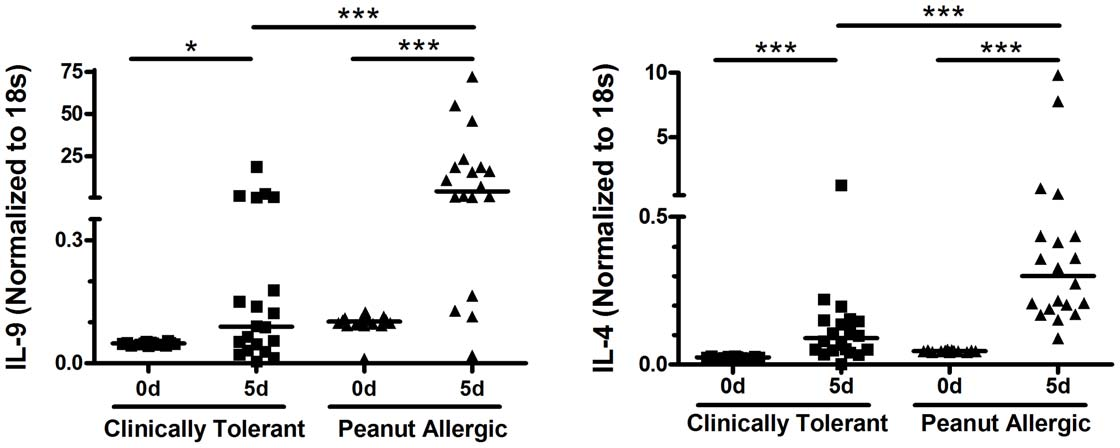
Antigen-dependent activation of cytokine mRNA expression. Matched pair analyses of IL-9 and IL-4 expression was assessed relative to 18 s rRNA in the presence and absence of peanut stimulation. Note break in y axis. n = 40. * p<0.05; *** p<0.0001.

**Figure 3:**
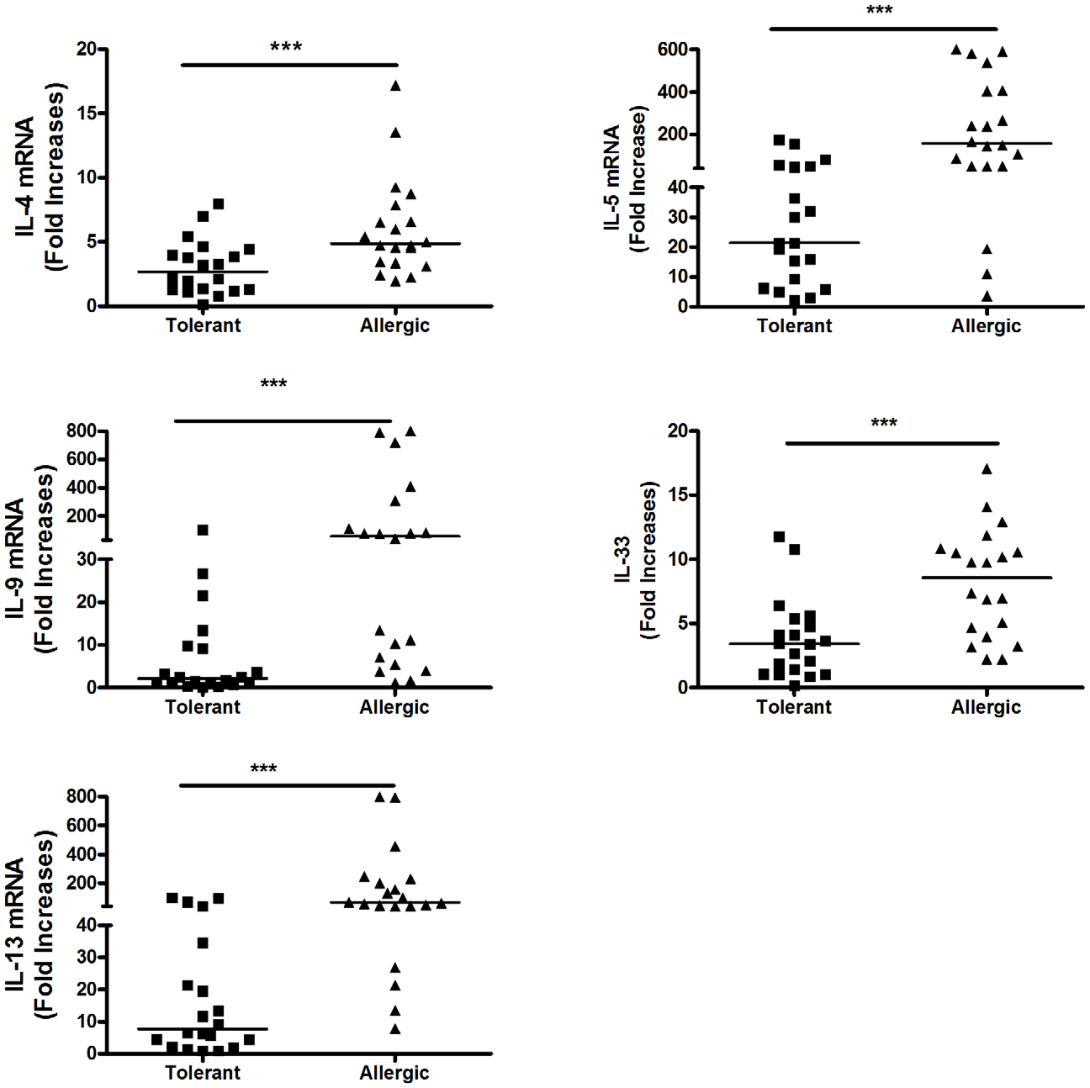
Comparison of peanut-dependent Type 2 mRNA profiles elicited in peanut allergic and peanut clinically tolerant individuals. Following in vitro PBMC stimulation (100 ug/ml), RNA was isolated from cells harvested at day 1 (IL-33), day 3 (IL-4), or day 5 (IL-5, IL-9, IL-13), times of optimal Ag-driven cytokine production. Results are expressed as fold-increased levels relative to matched un-stimulated controls. Bars indicate median responses. *** p<0.0001.

These conditions for short term Ag-driven stimulation of fresh cells in primary culture were used to seek qualitative or quantitative differences in cytokine responses between groups clinically tolerant to peanut versus those with clear clinical histories of allergic symptoms and signs after peanut ingestion. Fresh PBMC were cultured with peanut then cell pellets and supernatants harvested at the optimal time point for each candidate cytokine. In the absence of peanut stimulation, cytokine mRNA production ex vivo was usually extremely low in culture (Figure 2). As anticipated, upon peanut stimulation IL-5 was produced by virtually all peanut allergic individuals studied. Importantly, a high proportion of clinically tolerant individuals also exhibited significant responses (Figure 3). Median Ag-dependent IL-5 mRNA up-regulation in the clinically tolerant group was 21-fold (range: 0–174×) whereas the peanut allergic group had median 165-fold enhancement (range: 4–600×). This reflects an approximately eight times stronger intensity of IL-5 responses by peanut allergic individuals (ie 21-fold vs 165-fold increased, p<0.0005). IL-13 exhibited a similar pattern, with median 7.8 vs 66-fold increases upon Ag re-exposure, an 8.4× more intense response in the peanut allergic group (p<0.0001). Peak IL-4 mRNA levels (d.3) differed significantly between groups (p<0.0001, n = 40) but the intensity of that difference was quite limited (median 2.7 vs 4.9 fold increases, a 1.8 times differential). In contrast, IL-9 responses were discovered to be the most robust discriminator between clinical phenotypes with median 2.1 vs 59 fold enhancement in tolerant vs allergic groups, a 28 times more intense response by peanut allergics.

Assessment of IL-33 responses, a potent promoter of type 2 immune responses [19], [20], also revealed a strong association with clinical phenotype. Clinically tolerant individuals generated median 3.4 fold increases in IL-33 mRNA following peanut re-stimulation whereas responses in peanut allergic individuals were 8.6 fold enhanced. This 2.5 fold differential between phenotypes (p<0.0001) indicates that widespread and differential intensities of not only Th2-associated effector cytokines (IL-4, 5, 9 and 13) but also of cytokines associated with induction (or perpetuation) of allergic disorders is (i) a characteristic of peanut allergic individuals and (ii) occurs in both peanut allergic and peanut tolerant individuals upon exposure to this common food antigen.

**Figure 4:**
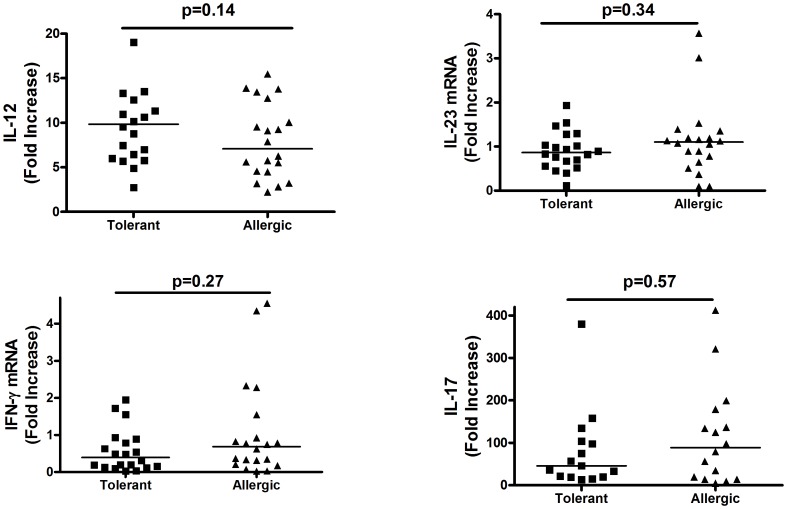
Peanut-driven stimulation does not elicit differential Th1/Th17-associated cytokine responses in peanut allergic vs. clinically tolerant individuals. PBMC were cultured as described for Figure 3 and comparisons of the intensity of the Ag-stimulated response (Wilcoxon) were made for each cytokine at the time of its peak antigen-driven response. Results are expressed as fold-increased levels relative to matched antigen un-stimulated controls. Bars indicate median responses. *** p<0.0001.

Th1 and Th17-immunity associated cytokines (IL-12, IL-23, IFNγ, IL-17), considered by some to be contributors to oral tolerance in food allergy, reveal a markedly different picture from that seen for type 2 immunity responses (Figure 4). Increased IL-12/23 p40 was detected relative to un-stimulated control cultures but the intensity of these responses did not differ between peanut allergic and tolerant populations. Enhanced IL-23 (p19) mRNA was seen in response to in vitro peanut stimulation in only two of 40 individuals examined. Similarly, median IFNγ mRNA levels were very low and did not differ between groups. Interestingly, while four (of 40) individuals did exceed the threshold of 2-fold enhancement in mRNA levels widely used to define a positive response, both were in the peanut allergic rather the clinically tolerant group. These data argue against a pivotal role for IFNγ in clinical tolerance to peanut.

IL-17 mRNA was strongly induced in both clinically tolerant and peanut allergic populations. As seen in Figure 4, comparable increases of 46 and 85 fold (medians, p = 0.57) were elicited following peanut stimulation. Thus, among the panel of cytokines assessed, no qualitative or quantitative differences were evident between clinically tolerant and allergic populations in pro-Th1/pro-inflammatory/Th17-like activation.

### Recall cytokine protein secretion in response to peanut antigen

**Figure 5:**
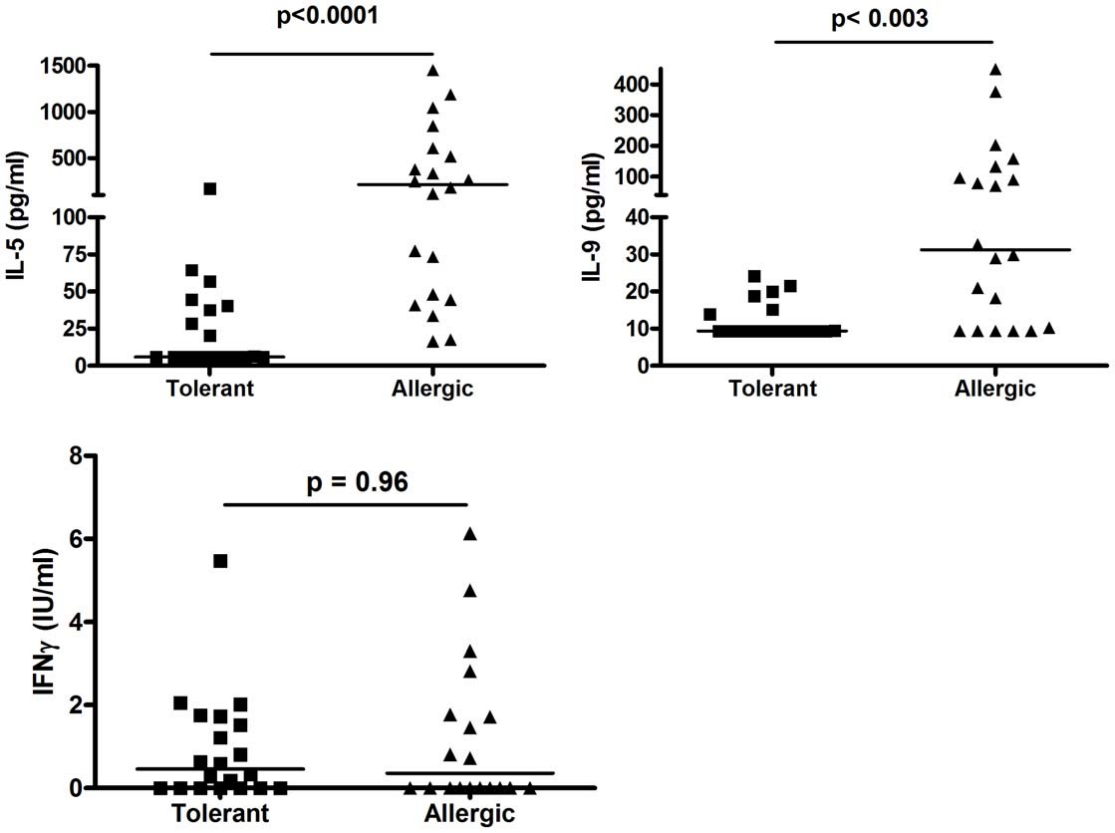
Cytokine protein profiles in culture supernatants of peanut allergic individuals and those exhibiting clinical tolerance to peanut. Culture supernatants harvested after five days of peanut Ag (100 ug/ml) stimulation were analyzed by ELISA, with median values shown for each population (Wilcoxon). In most instances, cytokine levels in unstimulated medium controls were at or below the limits of detection detailed at Materials and Methods.

Because mRNA expression and cytokine protein assessment have different experimental caveats, we quantified levels of representative cytokines in culture supernatants by ELISA. Elevated IL-5, IL-9 (Figure 5) production was readily demonstrable at the protein level, and recapitulated mRNA data. Similarly, as seen for mRNA levels, IFNγ protein levels elicited by peanut Ag were weak and indistinguishable between the two clinical phenotypes.

### Peanut- driven cytokine expression is Ag-specific

**Figure 6:**
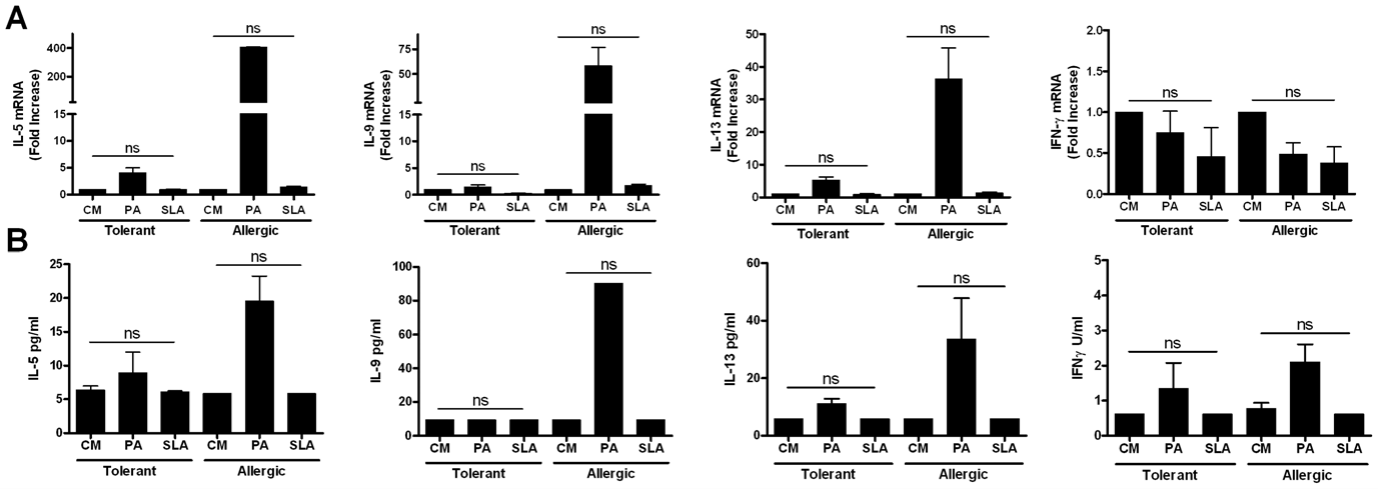
Antigen-specificity of mRNA expression. mRNA (panel A) and protein (panel B) responses elicited following culture in the presence of control medium, peanut Ag or soluble *Leishmania* antigen extract (SLA). Values given reflect responses of four individuals examined for each clinical phenotype.

**Figure 7:**
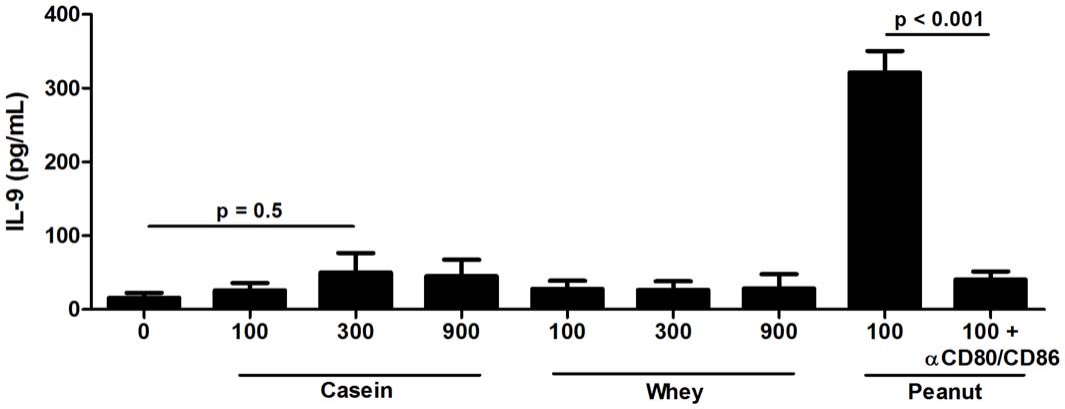
PBMC from peanut allergic individuals do not exhibit IL-9 responses to unrelated food antigens. Peanut stimulated IL-9 responses require costimulation. PBMC were stimulated for 5 days with 100 ug/ml peanut extract plus/minus anti-CD80/86 mAbs (each at 5 ug/ml) to assess costimulatory requirements for cytokine production *or* with unrelated the food antigen preparations bovine casein and whey at 100–900 ug/ml. Data reflect responses from triplicate analyses of culture supernatants obtained in nine independent experiments carried out using PBMC from highly peanut allergic (but milk non-allergic) individuals.

To confirm that the mRNA and cytokine protein responses quantified were truly peanut Ag dependent and not due to contaminants, polyclonal activators or spontaneous activation, we utilized three approaches. Endotoxin was ruled out as a contributor to cytokine production because LPS levels in both medium and peanut Ag were <0.01 EU/ml, well below the levels of LPS required to elicit production of these cytokines. Secondly, to assess peanut Ag-independent activation, we incorporated antigens that are highly stimulatory of cytokine production in exposed humans [21] but to which these volunteers had almost certainly not been exposed. Soluble *Leishmania antigen extract* (SLA) was used to stimulate PBMC from both groups, following which cell lysates and supernatants were harvested. IL-5, IL-9, IL-13 and IFN-γ mRNA and protein responses were quantified in eight independent experiments . Figure 6 demonstrates that these individuals respond to peanut but failed to produce any of these cytokines when stimulated with *Leishmania*. As a third approach, we recalled individuals with particularly strong peanut-stimulated IL-9 responses in order to assess the impact of stimulation with unrelated food antigens and, separately, of the capacity of in vitro addition of anti-CD80 and anti-CD86 Ab to block peanut stimulated IL-9 production. As shown in Figure 7, peanut extract stimulated IL-9 production is strictly dependent on costimulation. It was also found that peanut allergic and peanut non-allergic individuals (not allergic to milk) exhibited low level or undetectable IL-9 responses when stimulated with major milk allergens. Collectively these data highlight the antigenic specificity of the peanut-driven cytokine production assessed.

### Relative expression levels of cytokine mRNA

**Figure 8:**
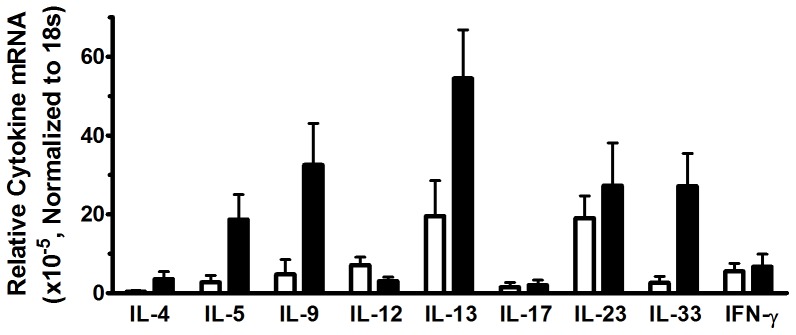
Relative utility of individual cytokine mRNA responses to differentiate between peanut allergic individuals and those with clinical tolerance to peanut. Total RNA was isolated from PBMC of peanut tolerant (open bars) and peanut allergic (closed bars) following in vitro stimulation with 100 ug/ml peanut extract for the time period leading to maximal Ag-driven cytokine production. mRNA levels were assessed by Q-PCR and are expressed relative to housekeeping gene 18 s rRNA rather than unstimulated medium controls, to enable comparison across cytokines, thereby identifying which cytokines exhibit the greatest differential response between peanut tolerant and peanut allergic populations.

The data presented above assess mRNA responses for each individual cytokine upon stimulation relative to expression of that cytokine in the absence of peanut antigen. To directly compare responses *between* cytokines, we calculated fold changes in mRNA after peanut stimulation, with each expressed relative to 18 s rRNA (Figure 8). As such, the data provide an indication of the relative utility of each cytokine as a potential tool to differentiate between populations with peanut allergy and those exhibiting clinical tolerance upon peanut exposure. . The cytokines exhibiting the most intense differential responses, hence greatest potential utility, when comparing clinical phenotypes were, in order, IL-9, IL-33, IL-5, IL-13 and IL-4.

## Discussion

Two important barriers to increasing understanding of the basic biology of food allergy have been a focus on a small number of prototypic cytokines in humans and the widespread use of antigen non-specific activation protocols to assess food allergen-specific human immune responses. While broadly similar, antigen-driven and polyclonally stimulated responses can yield very different conclusions about immune status [22]–[24]. To guide future experimental design into mechanisms that underlie food allergy vs clinical tolerance in humans, as well as to further spur development of novel multi-parameter diagnostic and management approaches for food allergy, we assessed a broad panel of cytokines characteristic of human Th1, Th2, Th9 and Th17-like functional phenotypes.

This is the first report of the importance of IL-9 in the human response to food allergens. Importantly, as for the other type 2 immunity cytokines examined here, its production in response to acute food Ag restimulation was not restricted to individuals who exhibit demonstrable clinical sensitivity upon peanut exposure. Indeed, each of the Th2/Th9-like cytokines was frequently stimulated by peanut re-exposure in peanut tolerant as well as in peanut allergic populations. Thus, just as the presence of food-specific serum IgE does not in itself reflect clinical sensitivity [25], expression of peanut-dependent Th2/Th9-like cytokine production in clinically tolerant humans is not indicative of clinical sensitivity. The capacity to generate and maintain cytokine production to this common food allergen is not inherently distinct between allergic and peanut tolerant humans as such responses are clearly ubiquitous in both populations. Rather, quantitative differences in the intensity of several of these responses offer potential for more compelling biomarkers. Given the many economic, logistical, safety and even interpretative concerns associated with food challenges [26], development of improved biomarkers continues to be a major goal [27].

For over two decades, IL-9 has been recognized as a cytokine whose elevated expression is linked to asthma and some related allergic disorders [28]–[29]. The extensive roles played by IL-9 in immune regulation [30] and effector mechanisms in humans are beyond the scope of this report but we note murine studies indicating that IL-9 can promote systemic anaphylaxis [31], [32]. Indeed, among all cytokines examined in the present report, that which exhibits most clear cut differential expression between clinical phenotypes was IL-9. More focus on this understudied cytokine may offer insight into immuno-regulatory events that distinguish food allergic from clinically tolerant humans. While cytokine selective therapeutic strategies have proven to be problematic in most studies [33], ongoing Phase II studies of the impact of blocking IL-9 function are underway in asthma [34].

This is also the first report of Ag-stimulated IL-33 in food allergy. IL-33 mediates its biological effects via ST2, a receptor selectively expressed on Th2 but not Th1 cells [19], [20], [35]. Here, IL-33 mRNA levels were found to differ quantitatively, with clinically tolerant individuals exhibiting a median 3.4 fold increase and allergic individuals 8.6 fold enhancement upon food Ag stimulation. This 2.5 fold differential in the intensity of responses between clinical phenotypes reveals that elevated production of cytokines associated with induction (or perhaps renewal) of allergic disorders, as well as that of classical Type 2 effector cytokines, occurs in both food allergic and non-allergic individuals, and at quantitatively distinct levels.

The role of pro-inflammatory responses in maintenance of clinical tolerance to food antigens remains controversial. Some groups argue for IFNγ in establishing and maintaining clinical tolerance to food antigens [36]–[38]. Others, including ourselves, argue that “protective” induction of pro-inflammatory cytokine production by food antigens would be highly hazardous and that alternative mechanisms of oral tolerance are more likely. Evidence exists for both perspectives. Here, upon examination of a panel of cytokines associated with induction (IL-12, IL-23) or expression (IL-17, IFNγ) of pro-inflammatory responses in humans, substantive and equivalent IL-17 mRNA responses were elicited by peanut in both groups, with undetectable levels of IL-17A protein by ELISA (data not shown). No evidence was found for peanut-dependent Th1-associated mRNA or protein production. By comparison, Reovirus, a ubiquitous viral agent that infects cells in respiratory and enteric tracts without causing clinically apparent disease, typically elicits IFNγ recall responses from 10 to >200 IU/ml when PBMC were cultured under virtually identical conditions [39]. Most importantly, none of the pro-inflammatory Th1 or Th17 associated cytokines are differentially induced in the groups studied. Thus while pro-inflammatory cytokines clearly play important, usually undesirable, roles in asthma and other allergic disorders [40], [41], neither elevated Ag-dependent Th1-associated nor Th17-associated activation appears to be associated with inhibition of food allergy in humans.

Multiple other pathways have been implicated in oral tolerance. Among these, elevated IDO, CTLA4/CD152, IL-10 and/or TGFb activity and enhanced Treg (of which multiple non-overlapping phenotypes and diverse mechanisms have been implicated ) are widely considered as key candidates [42]–[49]. None are the focus of this report. However, recent evidence (Lotoski et al, manuscript in preparation) indicates that IL-10 plays a key role in limiting the intensity of peanut Ag-driven cytokine responses among individuals with ongoing peanut allergy. Further studies on what mechanisms are responsible for development and maintenance of oral tolerance in humans exposed to ubiquitous food antigens are clearly needed.

An experimental limitation of this study applies to virtually all studies of human immune capacity to foods. Use is made of PBMC rather than gut derived lymphoid tissues. The ethical and logistical concerns that prevent gut biopsies in food allergic and healthy human controls, let alone in sufficient numbers of subjects with sufficient numbers of cells for functional studies in humans, are obvious. Aside from logistical arguments, there is also a good intellectual rationale for use of systemic cells to assess immune responses that are (sometimes) initiated in the gut. Food allergy is a systemic disease. Upon ingestion, food derived peptides have been reported to be systemically available [6]. Here, we find that systemic Ag-dependent responses are evident in a large proportion of healthy and food allergic humans. Clinical reactions by peanut allergic individuals are of course systemic, not restricted to the gut. Analysis of systemic immune responses is the best currently available tool to obtain insight in well powered groups as to how immune systems of food allergic and clinically tolerant individuals differentially respond to food re-exposure.

In summary, we identified optimal conditions for analysis of a broad panel of cytokines of relevance to food allergy in humans, directly ex vivo. While peanut antigen-dependent cytokine responses are common in the general population, clearly differential intensities of response are found for Th2/Th9-associated activation whereas Th1 or Th17-like responses are minimal. Ag-driven IL-9 responses offer a novel, more promising biomarker of dysregulated immunity to differentiate peanut allergic from peanut tolerant individuals. These findings will facilitate a broader approach to investigating immune (in)activation mechanisms linked to food allergy and clinical tolerance in humans.

## Supporting Information

Figure S1 **Determination of optimal peanut antigen concentrations for eliciting cytokine mRNA responses.** Mean mRNA levels in sextuplicate PBMC cultures are shown for three individuals following stimulation with 50 µg/ml (squares) 100 µg/ml (triangles) or 200 µg/ml (circles). Results are expressed as mean (± SEM) fold change in mRNA levels relative to expression at time zero in paired un-stimulated medium controls.
(TIF)

Table S1 **Primers validated for assessment of mRNA levels by Q-PCR.**
(DOCX)

Table S2 **Reproducibility of Replicate Q-PCR Analyses.**
(DOCX)
