# Supplementary figures and images for: Elevated Antigen-Driven IL-9 Responses Are Prominent in Peanut Allergic Humans
Source: PLoS One. 2012 Oct 11;7(10):e45377. doi: 10.1371/journal.pone.0045377 (PMC3469559; doi:10.1371/journal.pone.0045377)

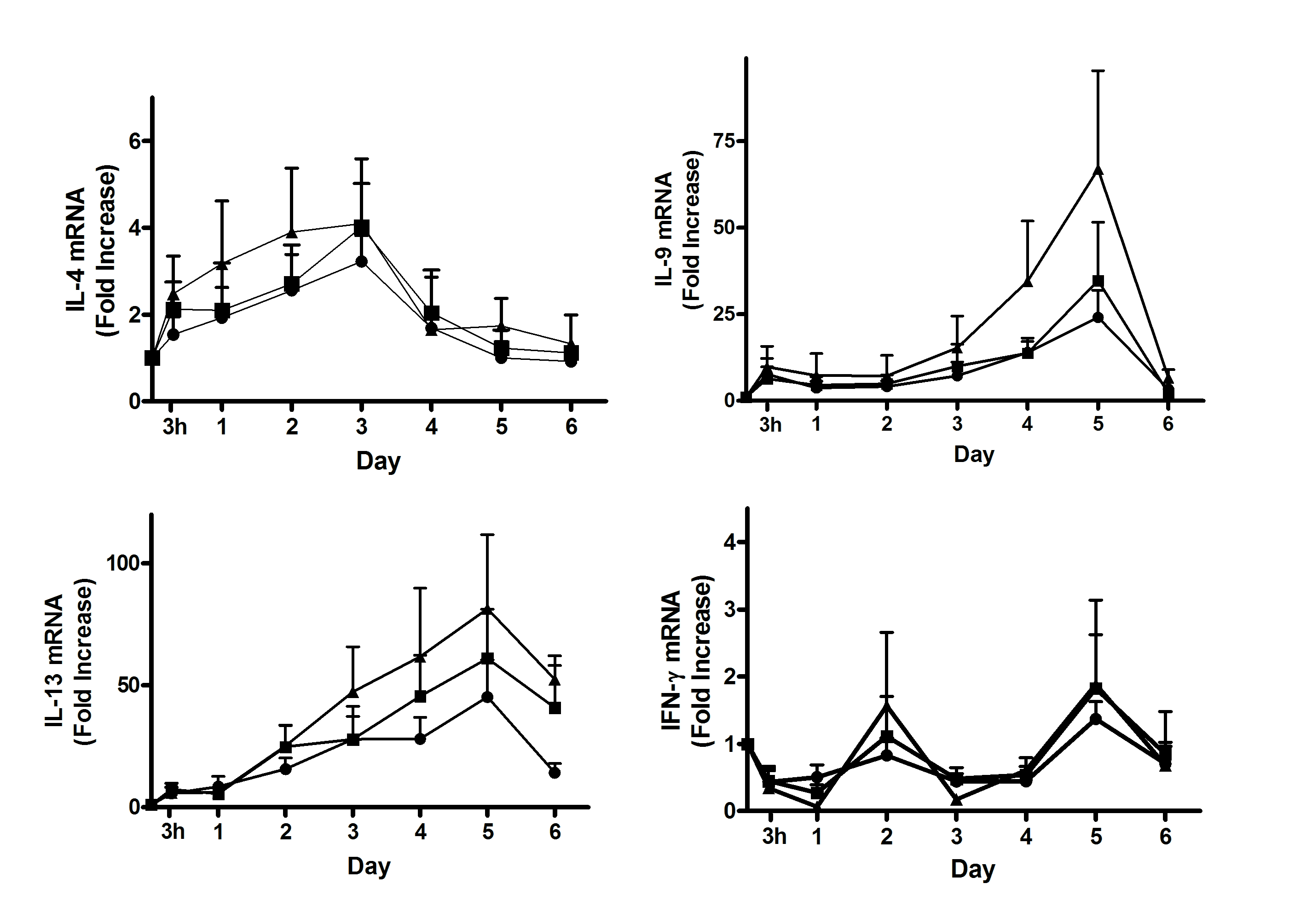

Supplement: Figure S1 — Determination of optimal peanut antigen concentrations for eliciting cytokine mRNA responses. Mean mRNA levels in sextuplicate PBMC cultures are shown for three individuals following stimulation with 50 µg/ml (squares) 100 µg/ml (triangles) or 200 µg/ml (circles). Results are expressed as mean (± SEM) fold change in mRNA levels relative to expression at time zero in paired un-stimulated medium controls. (TIF) [file pone.0045377.s001.tif]
