# Supplementary material for: Elevated Antigen-Driven IL-9 Responses Are Prominent in Peanut Allergic Humans
Source: PLoS One. 2012 Oct 11;7(10):e45377. doi: 10.1371/journal.pone.0045377 (PMC3469559; doi:10.1371/journal.pone.0045377)
Supplement: Table S1 — Primers validated for assessment of mRNA levels by Q-PCR. (DOCX) [file pone.0045377.s002.docx]

**Table S1:**

**Primers validated for assessment of mRNA levels by Q-PCR**

| *Gene* | Forward primer | Reverse primer |
| --- | --- | --- |
| *IL-4* | actgcacagcagttccacag | ctctggttggcttccttcac |
| *IL-5* | gagaccttggcactgctttc | ttcttcagtgcacagttggtg |
| *IL-9* | cctggacatcaacttcctcatc | tctggtgcagttgtcagagg |
| *IL-12 p35* | gaggcctgtttaccattgga | tactaaggcacagggccatc |
| *IL-12 p40* | cattgaggtcatggtggatg | caagttcttgggtgggtcag |
| *IL-13* | tgaggagctggtcaacatca | caggttgatgctccataccat |
| *IL-17A* | ccccagttgattggaagaaa | ttcgtgggattgtgattcct |
| *IL-23 p19* | tgggacacatggatctaagaga | ggatcctttgcaagcagaac |
| *IL-33* | caaagaagtttgccccatgt | aaggccttttggtggtttct |
| *IFN-γ* | gagtgtggagaccatcaagga | catgtattgctttgcgttgg |
| *18s rRNA* | ccgcagctaggaataatgga | ccctcttaatcatggcctca |
