# Supplementary material for: Elevated Antigen-Driven IL-9 Responses Are Prominent in Peanut Allergic Humans
Source: PLoS One. 2012 Oct 11;7(10):e45377. doi: 10.1371/journal.pone.0045377 (PMC3469559; doi:10.1371/journal.pone.0045377)
Supplement: Table S2 — Reproducibility of Replicate Q-PCR Analyses. (DOCX) [file pone.0045377.s003.docx]

**Table S2: Reproducibility of Replicate Q-PCR Analyses**

| **Subject** | **IL-9 mRNA (fold increase)** | | | | |
| --- | --- | --- | --- | --- | --- |
|  | ***1d*** | ***2d*** | ***3d*** | ***4d*** | ***5d*** |
| **A (first assay)** | **0.73** | **1.99** | **12.3** | **29.2** | **110** |
| **A (second assay)** | **0.98** | **2.48** | **15.7** | **35.8** | **139** |
| **B (first assay)** | **0.72** | **12.6** | **243** | **333** | **869** |
| **B (second assay)** | **1.92** | **14.1** | **233** | **367** | **877** |
